# Biobased production of alkanes and alkenes through metabolic engineering of microorganisms

**DOI:** 10.1007/s10295-016-1814-y

**Published:** 2016-08-26

**Authors:** Min-Kyoung Kang, Jens Nielsen

**Affiliations:** 1 Department of Biology and Biological Engineering, Chalmers University of Technology, Kemivägen 10, 412 96 Göteborg, Sweden; 2 Novo Nordisk Foundation Center for Biosustainability, Technical University of Denmark, Kogle allé, 2970 Hørsholm, Denmark; 3 Science for Life Laboratory, Royal Institute of Technology, 17121 Solna, Sweden

**Keywords:** Metabolic engineering, Alkanes/alkenes, Fatty acid biosynthesis, Cell factories, TRY (titer, rate, and yield)

## Abstract

Advancement in metabolic engineering of microorganisms has enabled bio-based production of a range of chemicals, and such engineered microorganism can be used for sustainable production leading to reduced carbon dioxide emission there. One area that has attained much interest is microbial hydrocarbon biosynthesis, and in particular, alkanes and alkenes are important high-value chemicals as they can be utilized for a broad range of industrial purposes as well as ‘drop-in’ biofuels. Some microorganisms have the ability to biosynthesize alkanes and alkenes naturally, but their production level is extremely low. Therefore, there have been various attempts to recruit other microbial cell factories for production of alkanes and alkenes by applying metabolic engineering strategies. Here we review different pathways and involved enzymes for alkane and alkene production and discuss bottlenecks and possible solutions to accomplish industrial level production of these chemicals by microbial fermentation.

## Introduction

Environmental concerns and depletion of fossil fuels have raised interest in producing biofuels and bio-based chemicals with an environmental footprint that is lower than current production from fossil fuels [29, 48, 65]. Until the early 2000s, metabolic engineering was applied primarily to improve titer and productivity of industrial fermentation processes [45]. To date, integration of synthetic biology and systems biology into the field of metabolic engineering have resulted in remarkable advancement of the field [16, 20]. With these advanced technologies it has become possible to produce by engineered cell factories more diverse compounds, which can be used as pharmaceuticals, chemical building blocks, and fuels [47]. In addition, we have acquired better understanding of complex cellular networks from the massive biological data sets provided by systems biology approaches and these findings have brought new tools and techniques that can be used to engineer cell factories.

**Fig. 1:**
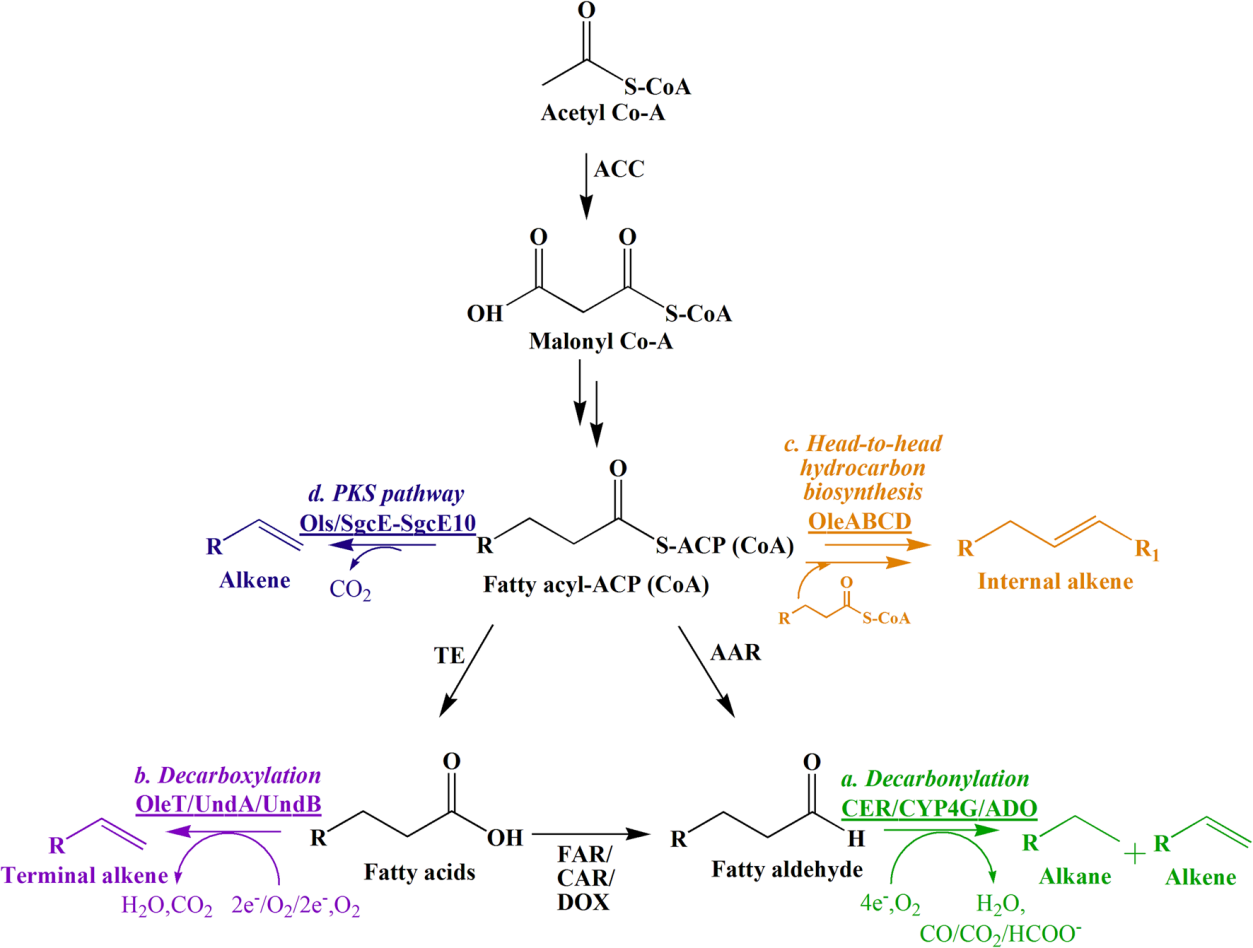
Alkane/alkene biosynthetic pathways and enzymes, which were utilized in previous reports. *a* Conversion of fatty aldehydes to alkanes/alkenes by AD enzymes, CER (plant), CYP4G (insects), and ADO (cyanobacteria), *b* terminal alkene production by decarboxylation enzymes, OleT, UndA, and UndB, *c* internal alkene biosynthesis by head-to-head hydrocarbon biosynthetic enzyme, OleABCD, *d* alkene production by PKS pathway enzymes, Ols and SgcE-SgcE10

Alkanes and alkenes are a very important class of hydrocarbons, used as liquid transportation fuels and as plastics. However, to obtain alkanes and alkenes with the right properties, cracking of crude oils are necessary. The complexity of this process can cause technical difficulties in obtaining specific molecules as well as it can increase processing costs [33]. Many organisms synthesize alkanes and alkenes naturally for protection against environmental threats [27, 30, 54], but the production level and structures of the compounds are not ideal for direct utilization as drop-in fuels. Several pathways and enzymes involved in alkane and alkene biosynthesis from natural producers have been discovered (Fig. 1) and introduction of the biosynthetic pathways into heterologous microbial hosts has allowed for production of various structures of alkanes and alkenes (Table 1). Many efforts have been made to produce alkanes and alkenes in engineered microbial strains, and recent advancements shows promise for possible future industrial production [47]. However, the titer, rate, and yield (TRY) of alkanes and alkenes production by heterologous hosts is still too low to meet industrial requirements and there are still several challenges that need to be overcome before these molecules can be produced by microbial fermentation.

**Table 1:** Examples of alkane/alkene production by metabolically engineered microorganisms

Enzyme	Strain	Products	Titer	References
AAR-AD	*E. coli*	Alkanes (C15, C17)	7.7 mg/L	[18]
	*E. coli*	Alkanes (C15, C17), Alkene (C17)	255.6 mg/L	[57]
	*E. coli*	Alkanes (C15, C17)	300 mg/L	[54]
	*E. coli*	Alkanes (C9, C12, C13, C14), Alkene (C13)	580.8 mg/L	[15]
	*S. cerevisiae*	Alkanes (C13, C15, C17)	22 μg/g of DW	[8]
	*S. cerevisiae*	Alkanes (C27–C31)	86 μg/g of DW	[6]
	*S. cerevisiae*	Alkanes (C13, C15, C17), Alkenes (C15, C17)	0.82 mg/L	[66]
	*Synechocystis* sp. PCC6803	Alkanes (C15, C17), Alkene (C17)	26 mg/L	[63]
FAR-AD	*E. coli*	Alkanes (C13, iso-C13, C15, iso-C15, C16, C17), Alkenes (C13, C15, C16, C17)	5 mg/L	[28]
CAR-AD	*E. coli*	Alkanes (C11, C13), Alkenes (C15, C17)	2 mg/L	[2]
DOX-AD	*S. cerevisiae*	Alkanes (C14, C16)	73. 5 μg/L	[24]
OleT	*E. coli*	Alkenes (C11, C13, C15, C15:2, C17:2)	97.6 mg/L	[41]
	*S. cerevisiae*	Alkenes (C11–C19)	3.7 mg/L	[10]
UndA	*E. coli*	Alkenes (C9–C13)	6 mg/L	[53]
UndB	*E. coli*	Alkenes (C5–C17)	55 mg/L	[52]
OleABCD	*E. coli*	Alkenes (27:3, 27:2, 29:2, 29:3)	40 μg/L	[5]
Ols	*Synechoccus* sp. PCC7002	Alkenes (C19, C19:2)	4.2 mg/L/OD_730_	[43]
	*S. globisporus*	Alkene (15:7)	129.3 mg/L	[14]
	*E. coli*	Alkene (C15)	140 mg/L	[40]

Here, we review the literature on alkane/alkene biosynthesis, and the associated fatty acid biosynthesis pathway and further discuss how the TRY challenge can be overcome to obtain efficient cell factories.

## Pathways and enzymes of alkane/alkene biosynthesis

### Decarbonylation of fatty aldehydes

Most pathways leading to alkanes/alkenes are via fatty aldehydes [36]. Aldehyde decarbonylases (ADs) have been discovered from various organisms including plants, insects, and cyanobacteria [6, 49] and they can convert fatty aldehydes to alkanes/alkenes with co-current production of carbon dioxide (CO_2_), carbon monoxide (CO), or formate depending on the organisms [42]. Some plants synthesize very-long-chain (VLC) alkanes to prevent water evaporation and protect themselves from environmental stresses [7]. The *Arabidopsis thaliana* aldehyde decarbonylase CER1 has been used to demonstrate alkane production in heterologous microbial systems [6, 15]. Insect cuticular layer is a mixture of alkanes/alkenes, and it acts as a barrier against environmental attacks as well as communication pheromones. Insect CYP4G1 is a P450 enzyme and co-expression of *Drosophila* CYP4G1 with cytochrome P450 reductase (CPR) enabled production of C23, C25 and C27 alkanes in *S. cerevisiae* [49]. Biosynthesis of alkanes has been identified in many cyanobacterial strains [54], but the reasons for alkane biosynthesis in cyanobacteria are unclear. ADs in cyanobacteria were initially reported to produce alkanes/alkenes and CO [54], but the actual co-product were later identified as formate by isotope tracer experiments. The cyanobacteria AD was therefore renamed as aldehyde deformylating oxygenase (ADO) [38].

There are two routes available for production of alkanes/alkenes via ADs, and these are from either fatty acyl-CoAs or from free fatty acids by the action of fatty acyl-acyl carrier protein (ACP) reductase (AAR) or fatty acid reductase (FAR), respectively (Fig. 1). *Arabidopsis* CER1 was revealed as an alkane biosynthetic enzyme through mutant and overexpression experiments in plants. However, alkanes were not detected when CER1 was solely expressed in yeast. Only through co-expression of Arabidopsis CER1–CER3 the production of VLC alkanes, chain-lengths C27–C31, was confirmed in yeast. In addition, alkane titer was increased and mainly resulted in production of nonacosane (C29) by 86 μg/mg of DW with co-expression of CYTB5s or/and LACS1, which acts as electron transfer components and long-chain acyl-CoA synthetase, respectively [6]. Even though CER1 produced VLC alkanes in yeast and plants, the enzyme was shown to enable production of C8–C14 alkanes in an *E. coli* strain that was engineered to produce short-chain fatty acids. The strain, that also encompassed several other engineering strategies, could produce short-chain alkanes up to 580.8 mg/L, and it was created by: (a) blocking the β-oxidation pathway by deleting the *fadE* gene which encodes acyl-CoA dehydrogenase, (b) increasing formation of short-chain FFAs by introducing modified thioesterase (TE), TesA with a L109P mutation and deleting the *fadR* gene, which is a transcriptional regulator, (c) expression of the *fadD* gene which encodes for fatty acyl-CoA synthetase for efficient conversion of FFAs to fatty acyl-CoAs, (d) expression of AAR from *Clostridium acetobutylicum* and CER1 from *A. thaliana* to produce short-chain alkanes from fatty acyl CoAs [15]. In an earlier study, cyanobacteria AAR activity was tested with two substrates, acyl-ACP and acyl-CoA in the presence of NADPH [54]. Both substrates were converted to fatty aldehydes by AAR, but acyl-ACP was the preferred substrate [54]. This enzyme has been frequently used for reconstruction of alkane/alkene biosynthetic pathways, and the expression of cyanobacteria AAR and ADO together in engineered strains results in production of predominantly C15 and C17 alkanes [8, 18, 54, 57, 63, 66]. However, the alkane titer in *S. cerevisiae* has not been reached the level of other hosts having co-expression of AAR-ADO. This was recently shown to be partly due to the presence of aldehyde dehydrogenase activity in yeast. Deletion of *HFD1*, encoding hexadecenal dehydrogenase (HFD), resulted in an increase in the alkane titer of *S. cerevisiae* by 22 μg/g of DW [8]. In addition, elimination of competing pathways (*Δpox1 and Δadh5*), increase of aldehyde supply (expression of carboxylic acid reductase (CAR) from *Mycobacterium marinum*), and enhancement of ADO expression (ADOs from *Synechoccocus elongatus* and *Nostoc punctiforme*) achieved an even highest alkane titer (0.82 mg/L) with an engineered *S. cerevisiae* [65], but it is still not comparable with the titers that can be obtained using *E. coli* (300 mg/L) [54].

Because fatty acids are abundant molecules in cells, they have been considered as desirable substrates for synthesis of alkanes/alkenes. Expression of the FAR complex from *Photorhabdus luminescens* [28], CAR from *Mycobacterium marinum* [2], and fatty acid α-dioxygenase (DOX) from *Oryza sativa* [24] enabled production of alkanes/alkenes when expressed together with AD. The FAR complex is encoded by reductase (*luxC*), synthetase (*luxE*), and transferase (*luxD*) gene operon, and it converts various chain-lengths of fatty acids into fatty aldehydes and expressed together with ADO from *N. punctiforme* it resulted in a more diverse range of alkane/alkene chain-lengths at a titer of 5 mg/L with *E. coli* compared with the use of the AAR-ADO pathway from *N. punctiforme* [28]. In addition, expression of FatB1, which is a C14 fatty acyl-ACP specific TE from *Cinnamomum camphora*, increased the C14 fatty acid and C13 alkane production in *E. coli* as well. The CAR enzyme has a broad substrate range (C4–C18), and addition of ATP and NADPH converts fatty acids to their corresponding fatty aldehydes, and co-expression of CAR and AD from *Prochlorococcus marinus* produce 2 mg/L of C11–C17 alkanes/alkenes in *E. coli* [2]. *O. sativa* DOX has the advantage as a fatty acid converting enzyme that it is using dioxygen as a co-factor, while AAR and CAR enzymes require NADPH and its substrate range is wider (C12–C18) than that of the cyanobacterial AAR (C16–C18).

### Decarboxylation of fatty acids

Terminal alkenes, often referred to as olefins, are important compounds in the chemical industry as they are used for the production of detergents, lubricants, and polyethylene. Three different types of enzymes, OleT_JE_, UndA, and UndB are involved in direct enzymatic conversion of fatty acids to terminal alkenes, and heterologous expression of these enzymes enabled production of terminal alkenes in engineered microbial strains [51–53]. OleT_JE_ is a cytochrome P450 enzyme belonging to the CYP152 family, and it was discovered from *Jeotglicoccus* sp. ATCC 8456. The CYP152 family OleT_JE_, forms alkenes by decarboxylation process rather than decarbonylation performed by the CYP4G family enzymes from insects. The CYP152 family members were reported to use only H_2_O_2_ as sole electron and oxygen donor, but recently H_2_O_2_-independent alkene biosynthesis was achieved using other biocatalytic co-factor systems: RhFRed, Fdr/Fdx, and CamAB [22, 41]. Recently the aldehyde decarboxylases, UndA and UndB, from *Pseudomonas* species were discovered, and both enzymes, when expressed in *E. coli*, resulted in production of terminal alkenes [52, 53]. UndA was found to be a non-heme iron oxidase, and the enzyme converted lauric acid (LA) to 1-undecene in the presence of Fe^2+^. In addition, it has a narrow substrate range, only medium-chain fatty acids (C10–C14) can be converted to ‘C-1’ corresponding terminal alkenes [53]. UndB was originally categorized as a fatty acid desaturase based on sequence homology, but found also to be an aldehyde decarboxylase. Compared with UndA, UndB shows a broad substrate range from C6 to C16, but it prefers C10–C14 fatty acids like UndA. The UndB homologue Pmen_4370 presented the highest conversion rate of undecanoic acid (C11) with *E. coli* strains co-expressing *E. coli* TE, UcFatB2 [52], but OleT provided the highest total titer of alkanes/alkenes in engineered *E. coli* strains (OleT: 97.6 mg/L, UndA: 6 mg/L UndB: 55 mg/L) [41, 52, 53].

### Head-to-head hydrocarbon biosynthesis

Nearly half a century ago, two research groups observed long-chain alkene biosynthesis in *Sarcina lutea*, but they could not elucidate the biochemical and genetic data in detail [3, 4, 61]. In a recent study of long-chain alkene biosynthesis by *Micrococcus luteus* resulted in identification of the three genes (Mlut_13230–13250) encoding OleABCD involved in alkane biosynthesis. Mlut_13230 has a conserved active site residue which is found in fatty acid biosynthesis enzymes, and its function as a OleA homologue was revealed by its ability to convert acyl-CoA to unsaturated monoketones in vitro [5]. Mlut_13240 and 13250 have sequence similarities with *oleD* and fusion of *oleB* and *oleC* from *oleABCD* in *Stenotrophomonas maltophilia* and heterologous expression of *oleABCD* from *M. lutea* resulted in production of 40 μg/L of long-chain alkenes in *E. coli.* In *Shewanella oneidensis* Strain MR-1, one of the long-chain alkenes, 3,6,9,12,15,19,22,25,28-hentriacontanonaene was synthesized, and the compound is interestingly identified from many bacteria which were isolated from cold environments. The deletion of the *oleABCD* homologue in the MR-1 strain resulted loss of alkane production supporting that the enzyme complex is involved in alkane biosynthesis [60].

OleABCD enzymes were specified based on extensive sequence homology analysis as a combination of a superfamily of enzymes consisting of thiolase (OleA), α/β-hydrolase (OleB), AMP-dependent ligase/synthase (OleC), and short-chain dehydrogenase/reductase (OleD) [59]. The proposed pathway of OleABCD is initiated by OleA, which involves a non-decarboxylative Claisen condensation to generate a β-keto acid and then OleD produces β-hydroxy acid by NADPH-dependent reduction [26, 59]. After the process, OleC converts the β-hydroxy acid to an alkene by consuming ATP [25]. Even though the role of OleB is unclear so far, a fusion of *oleB* and *oleC* in some organisms, propose a linkage to the activity of OleC or it is presumed to perform scaffolding or regulatory function in the Ole complex [62].

### Polyketide synthase (PKS) pathway

Polyketide and fatty acid biosynthetic pathways have very similar mechanisms, and both pathways are considered as promising for biofuel production. Generally, PKSs consist of 3-β-keto-acyl synthases (KS), acyl-transferases (AT), and acyl carrier proteins (ACP), β-keto reductases (KR), dehydratases (DH), enoyl reductases (ER), and finally a TE. The acyl substrates initiate the processes and malonyl-CoA extends the chain-length through elongation module, KS, AT, and ATP. After elongation the β-keto group is reduced to a β-hydroxyl group by KR, DH, and ER and then the TE domain catalyze decarboxylation and dehydration to release the alkene product. The *Synechococcus* sp. PCC 7002 synthesize C19 alkenes (1,14-nonadecadiene and 1-nonadecene) with terminal double bond, and to identify the involved enzyme, the sequence of Curacin A PKS was used to look for homologous potentially involved in alkane biosynthesis. CurM is the last module of Curacin A PKS from marine cyanobacteria *Lyngbya majuscule*, which forms the terminal double bond. Based on the sequence alignment result, one enzyme with 45 % amino acid homology to CurM was identified and it was named olefin synthase (Ols). In addition, Ols has several conserved domains of PKSs, loading domain (LD), ACP1, KS, AT, KR, ACP2, sulfotransferase (ST), and TE. To verify the involvement of Ols in long-chain alkene biosynthesis, an *ols* deletion mutant strain and an *ols* gene overexpression strain were constructed, and it was found that Ols deletion abolished alkene biosynthesis and strong expression of Ols increased alkene production by 4.2 mg/L/OD_730_ [43]. In another study, co-expression of enediyne PKS, SgcE and TE, SgcE10 from *Streptomyces* resulted in production of enediyne antibiotic C-1027 (37.5 mg/L) and 1,3,5,7,9,11,13-pentadecaheptaene (PDH, 129.3 mg/L) in engineered *Streptomyces globisporus* [14]. In addition, a SgcE-SgcE10 construct was introduced into *E. coli*, and it resulted in production of PDH. Through a chemical hydrogenation process, the cell culture extract product, PDH could be converted to pentadecane at a titer of 140 mg/L [40].

## Increasing TRY

Since the first report of alkane production in *E. coli* [54], there have been increased interests for engineering microorganisms to develop efficient cell factories that can be used for for alkane/alkene production in industry. Despite extensive knowledge on the metabolism of *E. coli* and *S. cerevisiae*, the two preferred cell factory platforms, the productivity of alkanes/alkenes by these organisms is much below what is required for industrial production. Costs of goods sold (COGS), around $0.6 per liter is considered to be the economically profitable value of microbial biofuel production. However, it is challenging to produce hydrocarbons at this value. For example, to produce pentadecane (C15) in *S. cerevisiae*, there is a requirement for NADPH and ATP that will reduce the yield on glucose. However, using detailed metabolic modeling the theoretical yield for production of different hydrocarbons in yeast, including alkanes/alkenes, and from this analysis it was found that even though the molar yield is lower than for ethanol the energy yield is only 5–10 % lower [9]. Furthermore, from a techno-economic analysis it was found that alkanes/alkenes, if 90 % of the maximum theoretical yield can be obtained, can be produced cost efficiently compared with ethanol [9]. A major factor for this is that costs of separation of hydrocarbons is likely to be lower than the relatively expensive distillation used for ethanol production.

However, it is clear that to meet the industrial feasibility, the microorganisms needs to be optimized in terms of TRY, and in the following we review the status on engineering cell factories for improving the TRY of alkanes/alkenes.

### Improving enzyme activities

Many enzymes have been discovered and applied to reconstruct the alkane/alkene biosynthesis in microorganisms, but they are commonly not very efficient and it is, therefore, difficult to reach high productivities. Since the last enzymatic steps such as decarboxylation and decarbonylation there is a loss of carbon in this step, and this impacts the product yield. This carbon loss is particular costly for production of short-chain alkanes/alkenes, whereas for longer chain alkanes/alkenes it is acceptable [9]. A major problem for increasing rate is, however, the low enzyme activity which will require high-level expression of the enzyme, something that may cause a significant protein burden for the cell. For instance, cyanobacteria ADO is a very slow enzyme and it cannot convert aldehydes efficiently to alkanes/alkenes, which generally results in accumulation of fatty alcohols as by-products [2, 8]. Fatty alcohols can be formed from fatty aldehydes by the action of unspecific alcohol dehydrogenases, of which there are many in both *E. coli* and *S. cerevisiae*. Therefore, researchers are trying to find better enzyme candidates from various resources by applying data derived from bioinformatics studies and sequence alignment analysis. In a previous study of OleT, several homologous genes of *oleT*_*JE*_ presented different productivity and distribution of chain-lengths, and a codon-optimized version of the best enzyme enabled increased production of alkenes [10]. Structure-based engineering of enzymes can also change the active site and hereby improve the enzyme activity. A modified ADO from *Procholorococcus marinus* with point mutation A134F had an altered substrate specificity with enhanced activity towards short-chain aldehydes and could hereby improve about twofold total titer of propane (0.46 mg/L) in *E. coli* [32].

Enzyme activities can also be optimized by changing the environmental condition. This was demonstrated by analysis of engineered *E. coli* strain carrying ADs from *Arabidopsis* and cyanobacteria. These enzymes have different optimal temperatures and CER1 expression was found to be strongest at 30°C and could at this temperature result in an alkane/alkene titer of 580.8 mg/L [15], whereas cyanobacterial ADO expression lead to the highest titer (26 mg/L) and cell mass (OD_600_ = 19.0) when the temperature was adjusted to 24°C compared with three other temperatures evaluated (18°C, 5.3 mg/L, OD_600_ = 8.5; 24°C, 5.0 mg/L, OD_600_ = 9.3; and 37°C, 4.2 mg/L, OD_600_ = 7.3) [57]. In the case of OleT co-expressed with CamAB and formate dehydrogenase (FDH), the optimal temperature for enzyme activity was different depending on the FA chain-length (highest activity at 4°C for C4–C9 and C18, and room temperature for C10–C16) [22]. As titer is an accumulated metric it is closely correlated with cell growth and the alkane/alkene titer was therefore found to increase with the use of rich media for engineered strains of *E. coli* expressing *Ado* and *S. cerevisiae* expressing *oleT* [10, 57].

### Improving precursor and co-factor supply

To increase the TRY of alkanes/alkenes, engineering of the fatty acid biosynthetic pathway is desirable as well as proper supply of acetyl-CoA, malonyl-CoA, and fatty acyl-CoA is important. Acetyl-CoA is a crucial molecule in microbial metabolism and it serves as one of the key precursors for fatty acid biosynthesis [35]. Because eukaryotic and prokaryotic cells have different acetyl-CoA metabolism, the strategies for precursor supply level increase depends on the cell factory platform used. In *S. cerevisiae* the acetyl-CoA metabolism is present in four different compartments (nucleus, mitochondria, cytosol and peroxisomes) and this makes it difficult to engineer acetyl-CoA metabolism in this cell factory. One strategy to enhance acetyl-CoA supply in the cytosol of *S. cerevisiae* was engineering of the pyruvate dehydrogenase (PDH) bypass in yeast, and this was achieved by expression of PDH from *Enterococcus faecalis* [34]. PDH is a very large multi-functional enzyme and it requires activation by lipoylation and as lipoic acid is synthesized in the mitochondria it was necessary to add lipoic acid to the medium [34, 46]. *E. faecalis* PDH has no mitochondrial targeting sequences unlike all eukaryotic PDHs, and it is relatively insensitive to high NADH but inhibited by high NADH/NAD^+^ ratios. Therefore, this PDH complex encoded by *pdhA, pdhB, aceF,* and *lpd* was able to function in the cytosol of *S. cerevisiae* and co-expression of *E. faecalis* lipolyation genes (*lplA* and *lplA2*) with the *E. faecalis* PDH enabled replacement of the native cytosol acetyl-CoA supply by ATP-independent mechanism [34]. In another strategy the ethanol degradation pathway was employed by overexpression of endogenous alcohol dehydrogenase (ADH2), acetaldehyde dehydrogenase (ALD6), and a mutant acetyl-CoA synthase (ACS with L641P) from *Salmonella enterica* [21]. Expression of these enzymes together with a wax ester synthase increased fatty acid ethyl ester (FAEE) production in *S. cerevisiae* [21]. Furthermore, this strategy also improved production of the plant sesquiterpen α-santalene in *S. cerevisiae* [13]. Compared to yeast, acetyl-CoA supply was designed differently in *E. coli* and cyanobacteria. In *E. coli* deletion of glucose fermentation pathway genes (*ΔadhE, ΔackA*-*pta, ΔldhA,* and *ΔfrdC*) redirected more carbon flux through acetaldehyde biosynthesis and overexpression of *S. enterica* EutE, a putative ALD/acetyl-CoA reductase, resulted in improvement of acetaldehyde production [68]. Improvement of alkane/alkene production was found in a *Synechocystis* mutant strain, which carried a deletion of the lactate biosynthesis gene, 2-hydroxyacid dehydrogenase (DDH), by overexpressing the AAR-ADO construct in the DDH site [63].

Malonyl-CoA is a key substrate for fatty acid biosynthesis and to malony-CoA production in is often increased by overexpression of acetyl-CoA carboxylase (ACC). It was reported that overexpression of endogenous *ACC1* alone increased the production of 3-hydroxypropionic acid, which is derived from malonyl-CoA, by 65 % in *S. cerevisiae* [12]. Wax ester production were also increased by 30 % when *ACC1* was overexpressed together with a wax ester synthase [55]. However, overexpression of *ACC1* does not always increase formation of products derived from the fatty acid biosynthetic pathways [10, 17]. To increase the fatty acyl-CoA pool, disruption of the β-oxidation pathway has been implemented in *S. cerevisiae* and through the deletion of *∆faa1* and *∆faa4,* the alkene titer was sevenfold increased [10]. In addition, deletion of additional genes involved in β-oxidation, *∆faa2, ∆pxa1, and ∆pox1* or *∆faa1, ∆faa2, ∆faa4, ∆fat1, ∆pxa1,* and *Δpox1* resulted in significant improvement of fatty acid production [37]. These strategies could reduce or completely eliminate competing pathways and hereby ensure more efficient production of fatty acids, and in particular ensure that there is no wasteful consumption of NADH and ATP, resulting in improved yield.

Together with precursor supply, proper co-factor supply can increase the productivity of alkanes/alkenes. Co-factor engineering has been used to ensure efficient OleT activity. OleT catalyze the decarboxylation of fatty acids to produce alkenes utilizing H_2_O_2_ as the sole electron and oxygen donor, and recently alternative systems was applied to replace the use of H_2_O_2_ [10, 22, 41]. In the presence of NADPH and oxygen, OleT used as redox partner the P450 reductase domain RhFRED from *Rhodococcus* sp. or the separate flavodoxin/flavodoxin reductase from *E coli* to produce alkenes [41]. Hem3, which is involved in heme biosynthesis was able to replace the use of H_2_O_2_ in alkene biosynthesis in *S. cerevisiae*, and it resulted in 8.7-fold increase of alkene titer by 427.7 μg/L [10]. Moreover, The CamAB components (CamA: NADH-putidaredoxin reductase and CamB: putidaredoxin) were able to convert fatty acids to alkenes when combined with a NAD(P)H regeneration system such as glucose dehydrogenase, phodphite dehydrogenase or FDH [22]. The ADs are required to utilize reducing components to increase alkane production and in yeast, VLC alkanes were improved when the CER1/CER3 were co-expressed with Cytochrome b5 (Cytb5) [6]. Similarly, co-expression of the insect CYP4G1 together with Drosophila CPR increased the production of alkanes in *S. cerevisiae* [49]. The important role of an efficient reducing system has been further supported by findings that expression of cyanobacteria ADO without a reducing system resulted in no alkane production and overexpression of reducing components from *E. coli* produced 2.7 μg/g of DW heptadecane in *S. cerevisiae* [8].

### Toxicity

Toxicity is a crucial issue to be considered when high productivity of chemical production is required in microorganisms. Alkanes/alkenes are toxic molecules in microorganisms, and change cell membrane integrity and function which cause inhibition of growth or even cell death [56]. To overcome chemical toxicity in microbial hosts, various strategies have been implemented. For example, overexpression of heat shock proteins or small regulatory RNAs, two-phase culture system, engineering of regulatory gene or enzyme, and these strategies all enhanced the solvent tolerance [31]. However, each strategy only partly solves the problem, and more basic research on the toxicity of alkanes is required. One approach is to combine toxicology and genomics, by some referred to as toxicogenomics, to map cellular responses to toxicants [1]. Recently, based on mechanistic understanding of cellular functions, transporters have emerged as targets for engineering in order to alleviate chemical toxicity [23, 44]. To investigate alkane/alkene related transporters in *S. cerevisiae*, the cells were exposed to C9–C12 alkanes, and several induced plasma membrane efflux pumps were identified from transcriptome data [39]. Some of the efflux pump candidates were verified, and overexpression of Snq2p and Pdr5p improved alkane tolerance by reducing intracellular decane and undecane concentrations [39]. In another study, ABC transporters in *Yarrowia lipolytica* were studied to identify efficient efflux pumps. Different chain-lengths (C8–C12) alkanes were supplied for susceptibility assay, and expression of ABC2 and ABC3 transporters increased the tolerance towards C10 and C11 alkanes [11]. In particular, ABC2 was revealed as the best efflux pump among four ABC transporters evaluated, and the reason for improved alkane tolerance was explained by a decrease in the intracellular alkane level. However, the efflux pumps did not improve C8 and C9 alkane tolerance and in *S. cerevisiae* overexpression of the Snq2p and Pdr5p efflux pumps only increased tolerance towards C10 and C11 alkanes but not towards C9 alkane [39]. This shows that efflux pumps are quite specific and it is, therefore, suggested to broaden the tolerance ranges by performing several strategies such as directed evolution and enzyme engineering by structure-based study [11, 39].

## Future outlook and conclusion

Alkanes/alkenes are industrially relevant chemicals, which can be used as starting materials in the chemical industry and as a liquid transportation fuel. Even though many organisms synthesize alkanes/alkenes naturally, industrial demands of these chemicals are currently only supplied by fossil fuels. Applications of the alkane/alkene biosynthesis in microorganisms can enable a future sustainable and green production of these important chemicals. Fatty acid biosynthesis provides several paths to re-construct alkane/alkene biosynthesis in heterologous microbial hosts. Efforts in microbial engineering for alkane/alkene production has shown that it is possible to engineer microorganisms for production of a diverse range of alkanes/alkenes. However, the TRY level of the compounds is far from the industrial requirements and further development is needed to obtain efficient cell factories.

For this intracellular biosensors may be valuable as they may allow for high-throughput screening and strain selection. Some of bacteria degrade alkanes/alkenes, and this has been exploited to create alkane biosensors. Alkane biosensors consist of three parts: alkane responsive transcriptional regulator, a promoter that is activated by regulator, and a reporter protein (e.g. GFP). Previous biosensors had problems for use in heterologous hosts because of limited detection range or proper function in a heterologous host [58, 67]. A new biosensor constructed as a chimera alkane response element (cARE), however, shows great promise [64, 65]. This was re-assembled using earlier biosensors, and enabled in situ detection of both mid- and long-chain alkanes by the fluorescence activated cell sorting (FACS) technology in *E. coli* [49, 65]. The use of a biosensor like this will enable faster screening of improved strains.

Even though it may be possible to produce alkanes/alkenes from sugar in a process similar to first generation ethanol production, our techno-economic analysis showed that using biomass as feedstock would be allow for further reduction of the production costs [9], and more importantly it will significantly reduce greenhouse gas emission, and even more than what is obtained with secondary bioethanol production. Therefore, it should be considered to reduce pre-treatment cost for lignocellulosic biomass and also engineer microorganisms for utilization of various carbon sources.

In conclusion, various natural alkane/alkene pathways have provided new enzymes that can be used for reconstructing alkane/alkene biosynthetic pathways in heterologous microbial hosts. Different combinations of these enzymes has allowed for increased production as well as enabled diversity of compound structures. Table 1 summarizes the titer of alkanes/alkenes in engineered strains, and it is observed that the AAR-AD pathway showed the highest titer among the reported biosynthetic pathways up to date. The enzymes showed different substrate specificities and activities. For example, UndB has broader substrate range than UndA [52], and also CAR from *P. marinus* synthesized several aldehydes while cyanobacteria AAR provide only two products [2, 54]. Besides, UndB displayed better conversion rate of fatty acids for alkene production compared with OleT and UndA [52]. It is, therefore, expected that discovery of new enzymes for alkane biosynthesis may allow for further improvement of alkane/alkene production. Furthermore, biosensors will probably promote the speed of enzyme screening and selection process, and biosensors will also enable faster evaluation of different strategies to improve the TRY of cell factories producing alkanes/alkenes and hereby reduce strain development time. In previous studies, engineered biosensors further enabled to control gene expression avoiding toxic intermediates accumulation in the cell [19], or it was possible to optimize individual reaction in biosynthetic pathways through real-time observation [50]. Together with such strategies, alkane/alkene biosensors will be possible breakthroughs to find the bottleneck steps faster, and also establish efficient cell factories by resolving current challenges in strain engineering.
